# Unveiling the Nanoconfinement Effect on Crystallization of Semicrystalline Polymers Using Coarse-Grained Molecular Dynamics Simulations

**DOI:** 10.3390/polym16081155

**Published:** 2024-04-19

**Authors:** Ji Yang, Yitong Chen, Zhangke Yang, Linjiale Dai, Hongseok Choi, Zhaoxu Meng

**Affiliations:** Department of Mechanical Engineering, Clemson University, Clemson, SC 29631, USA; jyang4@g.clemson.edu (J.Y.); yitongc@g.clemson.edu (Y.C.); zhangky@g.clemson.edu (Z.Y.); linjiad@g.clemson.edu (L.D.); hongc@clemson.edu (H.C.)

**Keywords:** semicrystalline polymers, nanoconfinement, coarse-grained molecular dynamics, heterogeneous crystallization

## Abstract

Semicrystalline polymers under nanoconfinement show distinct structural and thermomechanical properties compared to their bulk counterparts. Despite extensive research on semicrystalline polymers under nanoconfinement, the nanoconfinement effect on the local crystallization process and the unique structural evolution of such polymers have not been fully understood. In this study, we unveil such effects by using coarse-grained molecular dynamics simulations to study the crystallization process of a model semicrystalline polymer—polyvinyl alcohol (PVA)—under different levels of nanoconfinement induced by nanoparticles that are represented implicitly. We quantify in detail the evolution of the degree of crystallinity ($X_{C}$) of PVA and examine distinct crystalline regions from simulation results. The results show that nanoconfinement can promote the crystallization process, especially at the early stage, and the interfaces between nanoparticles and polymer can function as crystallite nucleation sites. In general, the final $X_{C}$ of PVA increases with the levels of nanoconfinement. Further, nanoconfined cases show region-dependent $X_{C}$ with higher and earlier increase of $X_{C}$ in regions closer to the interfaces. By tracking region-dependent $X_{C}$ evolution, our results indicate that nanoconfinement can lead to a heterogenous crystallization process with a second-stage crystallite nucleation in regions further away from the interfaces. In addition, our results show that even under very high cooling rates, the nanoconfinement still promotes the crystallization of PVA. This study provides important insights into the underlying mechanisms for the intricate interplay between nanoconfinement and the crystallization behaviors of semicrystalline polymer, with the potential to guide the design and characterization of semicrystalline polymer-based nanocomposites.

## 1. Introduction

Semicrystalline polymers constitute the largest fraction of synthetic polymers used for diverse engineering applications [1,2]. The properties of semicrystalline polymers depend on the unique internal structure with both crystalline and amorphous phases. The semicrystalline structures are intrinsically inhomogeneous and involve different length scales, ranging from molecules to nano-sized chain lamellae and micron-sized spherulites [2,3]. A fundamental understanding of the complex polymer crystallization affected by intrinsic and extrinsic factors can help to improve control over the properties of semicrystalline polymers and lead to novel inverse designs of polymer materials. The crystallization behavior of semicrystalline polymers is a classical problem of polymer physics research and is of great technological and scientific importance [4,5,6].

Particularly, polyvinyl alcohol (PVA) is a hydrophilic and biodegradable semicrystalline polymer and has been extensively studied [7]. PVA has excellent mechanical properties and resistance to both vapors and organic solvents [8]. Its biocompatibility allows PVA to blend with other biopolymer compounds [9,10,11]. Thus, it has many industrial applications, such as the mechanical property enhancement of other films [12,13,14], the field of textiles [15,16], and food packaging [17,18,19,20]. Similar to other semicrystalline polymers, the properties of PVA are greatly influenced by the processing conditions, which interplay with internal structures [21].

In the past decades, many studies have been conducted on the crystallization behaviors of PVA. The work done by Nikolaos and others investigated the kinetics of crystallization of solvent-free PVA and explored the crystallization mechanisms of PVA [22]. Recent studies also focused on the effect of nanofillers on the crystallization of PVA. For instance, Osamah and coworkers employed the Ozawa model and the Mo model to study the crystallization kinetics of PVA nanocomposites where graphene was used as the nanofiller. They found that graphene sheets lead to crystallite nucleation during crystallization [23]. Lee and co-workers conducted experiments to investigate the effect of polypyrrole nanoparticles (PPy NPs) on the crystallization behavior of PVA [24]. The results showed that PPy NPs enhance the overall degree of crystallinity. Hassen and co-workers concluded that the addition of nano-sized chromium oxide increases the degree of crystallinity of the PVA [25]. In contrast, Lee’s experimental work showed that PVA crystallization correlates with the concentration of silica nanoparticles [26]. Low concentrations of nanoparticles promote the crystallization of PVA, while higher concentrations would decrease the degree of crystallinity. Despite extensive experimental work, the influence of nanoparticles on the crystallization of PVA remains inconsistent and sometimes contradictory to each other.

Additionally, many experiments have been conducted to examine the impact of nanofiller-induced nanoconfinement on the crystallization process of various polymers. Bosq and Aht-Ong investigated the non-isothermal crystallization behavior of poly(butylene succinate) (PBS) nanocomposites blended with NaY zeolite nanoparticles [27]. The results showed that the addition of nanoparticles increased the crystallinity of PBS. Papadopoulos and coworkers studied the crystallization of polylactide (PLA) by adding different nanofillers [28]. The experimental results showed that nanofillers enhanced the crystallization rate, but the final degree of crystallinity was not necessarily increased. This study also showed that adding nanofillers led to significant alternations in the semicrystalline morphology. Another experimental study showed that the nanofillers could function as nucleation agents and influence the overall crystallinity of the PLA [29]. In contrast, Reinsch and Ludwig’s work showed that the crystallization rate of poly(ethylene terephthalate) is not affected by adding nanofibers [30]. These experimental studies have yielded inconsistent outcomes about the influence of nanoparticles on the crystallization of polymers.

Although the inconsistencies in experimental results can arise from variations in measurement methods and the types of nanoparticles used in different experiments, experimental work alone usually fails to offer a fundamental understanding of how nanoconfinement induced by diverse nanofillers influences the local crystallization behaviors of semicrystalline polymers. The interfaces between nanofillers and polymer chains serve as strong nanoconfinements that significantly influence chain dynamics [31,32]. Previous studies have shown that nanoconfinement from interfaces can influence the elasticity and other thermomechanical properties of polymer systems [33,34,35]. Nevertheless, for the non-equilibrium process of polymer crystallization, experimental investigations usually fall short in considering the effect of nanoconfinement on the local crystallization processes.

In contrast, computational methods serve as a powerful tool to fundamentally unravel the complex effect of nanoconfinement on the physical properties of polymers, including the crystallization process. Ming and coworkers utilized dynamic Monte Carlo (MC) simulations to investigate the crystallization behavior of confined polymer systems by considering factors including molecular weights, interface interactions, and lateral sizes [36]. However, MC simulations are not able to provide detailed trajectory information on individual molecules through time evolutions [37], thus hindering the delineation of crystallization process and kinetics. In comparison, molecular dynamics (MD) simulation shows a unique advantage as it can capture the dynamics of polymer chains at very fine temporal resolutions [38]. Previous efforts have used MD simulations to understand the influence of nanoconfinement on the thermomechanical properties of amorphous polymer systems [39,40,41]. Additionally, MD simulations have been previously applied to study polymer crystallization [42,43,44,45,46]. However, only a small number have investigated the crystallization of polymers under nanoconfinement. Jabbarzadeh investigated the effect of gold nanoparticles on the crystallization of polymers using large-scale MD simulations, and the results showed that the nanoparticles decreased the overall degree of crystallinity [45]. Han and co-workers studied the crystallization of polyethylene grafted onto carbon nanotubes by using MD simulation, and the results showed that the final crystallinity of the polymer increased with larger grafting density [46]. These two atomistic MD studies were limited in the spatiotemporal scale they could reach. In comparison, coarse-grained (CG) MD simulations show a unique advantage in simultaneously conserving essential molecular features and reaching greater spatiotemporal scales compared to atomistic simulations [47,48]. Moreover, previous studies mainly focused on the effect of explicit nanofillers on the overall crystallization of polymers. Nevertheless, the influence of nanoconfinement (independent of nanofiller shape and size) on the local crystallization behavior in the interphase region next to the interfaces and the associated crystallization kinetics, such as where and how nucleation happens, have remained largely unknown.

We aim to fill these knowledge gaps in this study by employing a well-established CG model for PVA to explore the local polymer crystallization behavior with strong nanoconfinement induced by nearby nanoparticles. Specifically, the CG model matches the structural properties of PVA from all-atomistic simulations [49] and has been shown to capture the semicrystalline features, mechanical responses, and deformation mechanisms [50,51,52,53,54,55]. Different from previous studies, our study focuses on how local nanoconfinement influences the crystallization process and kinetics of semicrystalline PVA. Specifically, we carry out MD simulations and investigate region-dependent crystallinity of bulk polymers and polymers under nanoconfinement induced by implicit nanoparticles. We also carefully examine the differences in the onset of crystallite nucleation. The cooling rate factor will also be considered in this study. Our results will provide insights into the detailed crystallization process in the local regions under different levels of nanoconfinement.

## 2. Materials and Methods

### 2.1. CG Model of PVA

**Figure 1:**
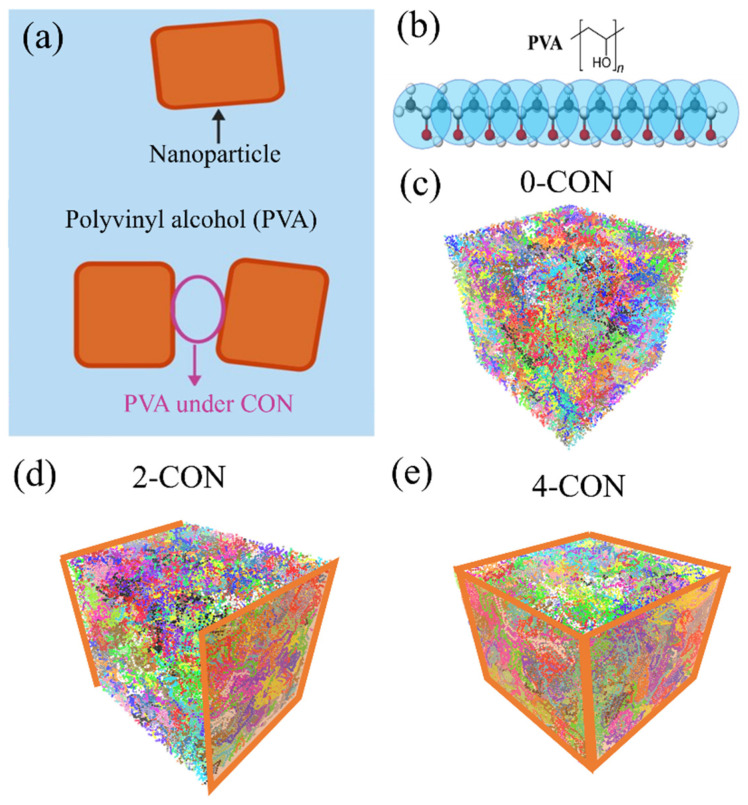
(**a**) A schematic of PVA under nanoconfinement induced by nanoparticles that we aim to study using our model systems. (**b**) Schematics of the CG model of PVA, where each CG bead (light blue) represents a PVA monomer. (**c**) Computational model of bulk PVA under no nanoconfinement (0-CON). (**d**) Computational model of PVA under double confinement (2-CON). (**e**) Computational model of PVA under nanoconfinement in four directions (4-CON). In (**d**,**e**), the orange planes illustrate the applied implicit rigid walls that represent nanoparticle surfaces, confining nearby PVA chains.

We apply the CG model of PVA to study the effect of nanoconfinement induced by nanofillers/nanoparticles on the crystallization process of PVA, as illustrated in Figure 1a. In the CG model of PVA, each constituent bead represents one monomer of a PVA chain [51,56], as shown in Figure 1b. The constituent beads are connected by bonds, while angles are formed between three successive beads. The force field of the CG model uses Lennard-Jones (LJ) units. Specifically, the potential expression of the bond used in this CG model is shown below:

(1) $$E_{bond}=k{\left(r-r_{0}\right)}^{2}$$

where k is the bond stiffness with k=1352, r is the distance between the two bonded beads, and $r_{0}=0.5$ is the equilibrium bond distance [51].

The angles are defined by a tabulated potential, derived from matching the angular distribution of atomistic simulations through the inverse Boltzmann method. The potential data and the implementation method can be found in the original work [51].

The non-bonded interactions between the CG beads are accounted for by the 9-6 style LJ potential expressed by:

(2) $$E_{nb}\left(r\right)=4\varepsilon_{nb}[{\left(\frac{\sigma_{nb}}{r}\right)}^{9}-{\left(\frac{\sigma_{nb}}{r}\right)}^{6}]\;\;\;\;\left(r\leq \sigma_{nb,c}\right)$$

where r is the distance between two non-bonded CG beads; $\varepsilon_{nb}=0.37775$ represents the depth of the potential well; $\sigma_{nb}=0.89$ is the distance at which $E_{nb}$ crosses zero. This potential is further shifted in the implementation so that its value becomes zero at the cutoff distance, $\sigma_{nb,c}=1.02$. All the bond, angle, and non-bonded potential parameters in our simulation are consistent with the original model [51,53].

### 2.2. Nanoconfinement Representation

We employ the Large-scale Atomic/Molecular Massively Parallel Simulation (LAMMPS) software (23 June 2022 version) to carry out all the MD simulations in this study [57]. The simulation trajectories are visualized using the Visual Molecular Dynamics software (29 June 2021 version) [58].

The computational domain of the PVA model comprises a hexahedral representative volume element (RVE) (40×40×40 in LJ unit), with a total of 200 chains and 500 monomers in each chain. These chains are represented by different colors in Figure 1c–e. We apply implicit rigid walls (illustrated by the orange planes in Figure 1d,e), which interact with PVA chains like nanoparticle surfaces, to represent the nanoconfined cases by using the ‘fix wall/lj126′ command implemented in LAMMPS. In comparison to a bulk PVA case with no such walls, named 0-CON (Figure 1c) in this study, we use double-confinement, i.e., 2-CON (Figure 1d), and quadruple-confinement, i.e., 4-CON (Figure 1e), cases to study different levels of nanoconfinement and how they influence the crystallization process of PVA. The interaction between the wall and PVA chains is modeled by the 12-6 style LJ potential:

(3) $$E_{wall}=4\varepsilon_{wall}[{\left(\frac{\sigma_{wall}}{r}\right)}^{12}-{\left(\frac{\sigma_{wall}}{r}\right)}^{6}]\;\;\;\;\;\;(r\leq \sigma_{wall,c})$$

where r is the normal distance from PVA beads to the wall; $\varepsilon_{wall}$ represents the depth of the potential well; $\sigma_{wall}$ is the zero potential distance; $\sigma_{wall,c}$ is the cutoff distance and equal to $3\sigma_{wall}$. In our simulation, we use $\varepsilon_{wall}=1$ and $\sigma_{wall}$= 1 (both in LJ unit), similar to those used between nanoparticles and polymer chains in previous computational studies on model polymer nanocomposites and specifically the interfacial zone around nanoparticles [59,60,61].

### 2.3. Simulation Procedures

The timestep used in the simulations is 0.01 in the LJ unit, which is equivalent to 0.0163 *ps.* Periodic boundary conditions (PBCs) are applied to the directions where no implicit rigid walls are present. For nanoconfined cases where rigid walls are applied accordingly, shrink-wrapped boundaries for the corresponding directions are used.

In the first stage of the whole simulation, energy minimization is carried out with the “soft” pair potential until the energy or force tolerance, or the maximum number of iterations or force/energy evaluations are reached. After this minimization process, the two rigid walls in the y direction are placed at ylo=−5 and yhi=45 for the 2-CON case to avoid direct overlap with PVA beads. For the 4-CON case, another two rigid walls in the z direction are positioned at zlo=−5 and zhi=45. Then, the system is run under the “nve/limit” ensemble for 30,000 steps. After that, the “soft” pair potential is replaced with the actual 9-6 style LJ pair potential (Equation (2)), and the energy minimization process is conducted again with this pair setting. Afterward, the NPT ensemble is applied in the following simulation stages. Under the NPT ensemble, the system first goes through a biaxial compression process as we move the rigid walls from their original positions to ylo=0, zlo=0 and yhi=40, zhi=40, respectively. This step makes sure that the bulk case and nanoconfined cases share the same initial cubic shape and sizes. After this step, the rigid walls are fixed to their places, i.e., no movement is allowed through the simulation. Then, the simulation is run at a constant temperature of T = 1 (corresponding to 550 *K* in real unit), at which PVA is at melt state, for $10^{6}$ time steps to fully equilibrate the PVA system. The temperature is controlled by the Nosé–Hoover thermostat, and the damping parameter is 100 timesteps. Meanwhile, the pressure is kept constant at 8 (corresponding to 1 atm) in directions where there are no walls present through a Brendsen barostat with the damping parameter as 1000 timesteps.

**Figure 2:**
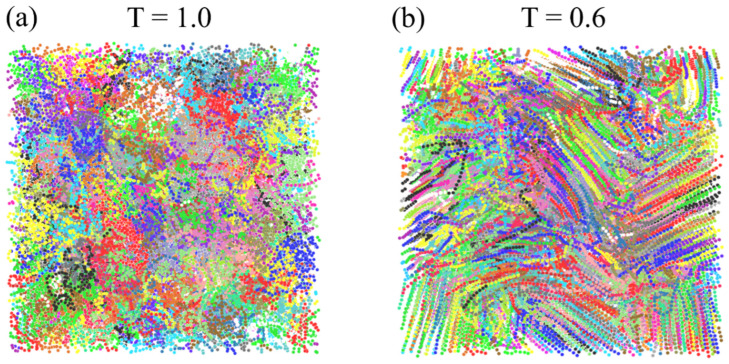
(**a**) The initial amorphous structure at T = 1, and (**b**) the semicrystalline structure at T = 0.6 for the 0-CON system.

Afterwards, we implement a cooling down process to initiate the crystallization process of the PVA. Through $4\times 10^{7}$ steps (i.e., cooling rate of $1\times 10^{-6}$ per timestep), the temperature of the system decreases from T = 1 to T = 0.6 while the pressure remains the same. We also apply different cooling rates by altering the total steps for the cooling down process. As shown in Figure 2, a semicrystalline structure, where many chains are folded back and forth, forming crystalline regions while still some amorphous regions exist, is formed at T = 0.6, after cooling down from the amorphous melt state structure at T = 1.

To calculate the degree of crystallization ($X_{C}$) for PVA in different states, we employ both $p_{2}$ and nematic order method. Many previous studies used the nematic order method to characterize the $X_{C}$ of semicrystalline polymers [53].

**Figure 3:**
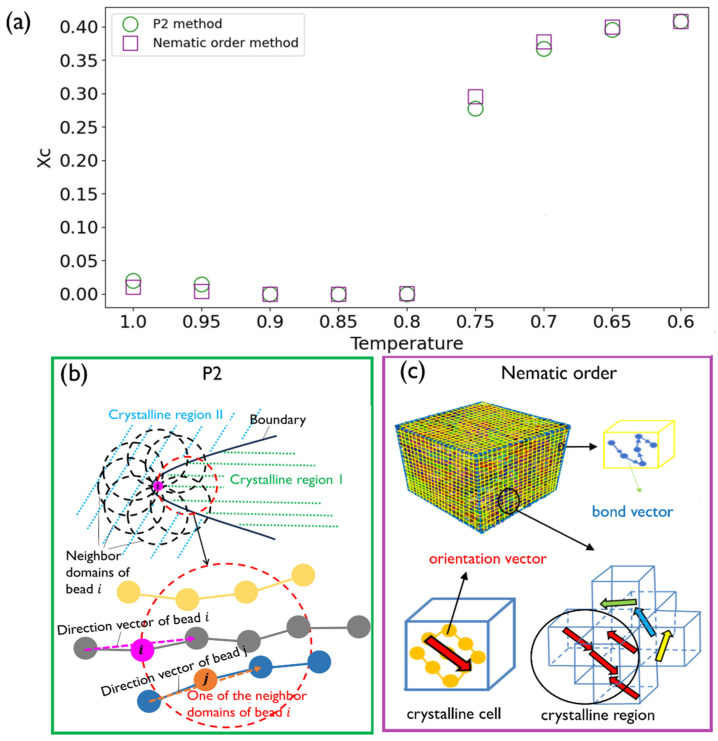
(**a**) $X_{C}$ calculated by either $p_{2}$ or nematic order method vs. temperature for the 0-CON system. (**b**) The 2-D demonstration of the calculation of $P_{2}$ with a group of neighbor domains. (**c**) Schematics showing the determination of crystalline regions based on the nematic order method.

In this study, we further propose that the $p_{2}$ method, also known as Herman’s order parameter [62,63,64], can be used to calculate $X_{C}$ of semicrystalline polymers with high efficiency and accuracy. We validate the accuracy of $p_{2}$ method predicted $X_{C}$ by comparing it to the well-established nematic order method in Figure 3a during the cooling down process of the 0-CON system depicted in Figure 2. The calculated $X_{C}$ values from both methods are consistent, demonstrating the validity of the $p_{2}$ method proposed in this study. Details of these two methods will be discussed next.

### 2.4. $p_{2}$ Method to Characterize $X_{C}$

The $p_{2}$ method is employed in this work to quantify the extent to which the polymer chains are ordered. The bead-wised $p_{2}$ value of the ith bead is calculated using the Equation (4) below [65]:

(4) $$p_{2}(i)=\frac{1}{N_{i}}\sum_{j=1}^{N_{i}}\left(\frac{3\cos^{2}\theta_{ij}-1}{2}\right)$$

where $N_{i}$ is the number of beads within the neighbor domain of the ith bead, excluding the beads that are on the same chain with the ith bead. $\theta_{ij}$ is the angle between the direction vector of the ith bead, $\vec{d}(i)$, and the direction vector of the jth bead, $\vec{d}(j)$, with $\theta_{ij}$ calculated by:

(5) $$cos\theta_{ij}=\frac{\vec{d}\left(i\right)\cdot \vec{d}\left(j\right)}{\left|\vec{d}\left(i\right)\right|\left|\vec{d}\left(j\right)\right|}$$

The direction vector of a bead is defined by the vector that points from one of its bonded beads (typically the one with a smaller id) to the other one. For example, the direction vectors of the ith and jth beads are, respectively,

(6) $$\vec{d}\left(i\right)=\vec{r}\left(i+1\right)-\vec{r}\left(i-1\right)$$

(7) $$\vec{d}\left(j\right)=\vec{r}\left(j+1\right)-\vec{r}\left(j-1\right)$$

where $\vec{r}$ is the position vector of the bead.

In previous studies [64,65,66], the neighbor domain of a bead i is defined as a spherical space centered at the bead i with a specified radius, $r_{c}$. We found that the $p_{2}$ value calculated based on this neighbor domain setup could underestimate the degree of order for the polymer chains near the boundary between two crystalline regions or between the crystalline region and amorphous region. To address this issue, we propose a new setup of neighbor domains that consist of a group of domains instead of just a single domain. Taking a two-dimensional case as an example, the $p_{2}$ value of the bead i, as shown in Figure 3b, will be calculated based on trying different neighboring circle domains, respectively. In three-dimensional cases, those domains are spherical regions with the same radius but centered at different positions. Basically, the centers of those neighbor domains follow such a pattern:

(8) $${\vec{r}}_{c,m}={\vec{r}}_{i}+r_{c}{\vec{n}}_{m}\;\;\;\;\;\left(m=1,2,\ldots ,N_{d}\right)$$

where ${\vec{r}}_{c,m}$ is the position vector of the center of the mth spherical neighbor domain of bead i, ${\vec{r}}_{i}$ is the position vector of the mth bead, $r_{c}$ is the radius of the spherical domains, ${\vec{n}}_{m}$ is the unit orientation vector, and $N_{d}$ is the number of neighbor domains for each bead.

The $p_{2}$ value calculated for bead i based on the mth neighbor domain is denoted as $p_{2}(i,m)$, $m=1,2,\ldots ,N_{d,i}$. We select the largest $p_{2}$ value as the final $p_{2}$ value of bead i, i.e.,

(9) $$p_{2}\left(i\right)=\max\left\{p_{2}\left(i,m\right)\right|m=1,2,\ldots ,N_{d}\}$$

Selecting the greatest $p_{2}$ value is justified in the schematic in Figure 3b, where only one specific domain or small set of domains realistically encompasses the same crystalline region for beads located at the region boundaries. In this work, 14 spherical neighbor domains are used for each bead, i.e., $N_{d}=14$. The corresponding unit orientation vectors to the neighbor domains are listed in Table 1. The radius of each spherical neighbor domain is chosen as 2.

Other choices of neighbor domains may achieve similar results so long as all orientations are covered by them appropriately. The selection of the neighbor domains employed in this work is a tradeoff between computational efficiency and accuracy. It turns out that the selected neighbor domains are able to give reasonable $p_{2}$ values that closely reflect the order of polymer chains, especially near the crystalline boundaries.

To calculate the $X_{C}$ of the whole system, the RVE of the system is divided into 8000 equal cubic cells, each having the size of 2×2×2 in LJ unit. Then, the $p_{2}$ value corresponding to each cell is defined as the average of $p_{2}$ of the beads in that cell. A cell is categorized as a crystalline cell if its $p_{2}$ value is greater than 0.8. After all the cells are examined by this criterion, the $X_{C}$ of the whole system is calculated as the volume fraction of crystalline cells within the RVE. We note that the obtained $X_{C}$ values for amorphous PVA at temperatures higher than 0.8 in Figure 3a are not exactly 0 but very close to 0. We believe these very small values are probably due to minimal possibilities where chains are aligned in a very small region. We believe these minor values tend to be random and they do not affect the major trends observed in this study.

### 2.5. Nematic Order Method to Characterize $X_{C}$

We also apply the nematic order method to calculate the $X_{C}$ of the system to validate the $p_{2}$ method. Specifically, the whole computational domain is equally divided into 20×20×20 small cubic cells, as depicted by Figure 3c, consistent with the cell sizes using $p_{2}$ method. A nematic tensor is then calculated for each cell using the directions of bonds within it [67]:

(10) $${\tilde{Q}}^{\left(i\right)}=\sqrt{\frac{3}{2}}\frac{1}{N^{\left(i\right)}}\sum_{j=1}^{N^{\left(i\right)}}\left({\vec{n}}^{\left(i,j\right)}⨂{\vec{n}}^{\left(i,j\right)}-\frac{1}{3}\tilde{I}\right)$$

where ${\tilde{Q}}^{\left(i\right)}$ is the nematic tensor of the ith cell, $N^{\left(i\right)}$ is the number of bonds inside the ith cell, ${\vec{n}}^{\left(i,j\right)}$ is the unit direction vector of the jth bond within the ith cell, and $\tilde{I}$ is the second-order identity tensor.

The largest eigenvalue of ${\tilde{Q}}^{\left(i\right)}$ defines the order parameter S for the ith cell, while the corresponding eigenvector represents the preferred orientation vector ${\vec{e}}^{\left(i\right)}$ of the ith cell [53]. The $X_{C}$ of the system is defined as the volume fraction of the cells whose order parameter S are greater than 0.8, similar to previous studies [53]. Again, the very small values at T≥0.8 in Figure 3a are minor calculation variances due to the small cell sizes used herein.

Additionally, we can recruit different crystalline unit cells (i.e., S>0.8) with the same preferred orientation vectors into crystallized domains or crystallites by using the nematic order method. In practice, if the cosine value of the angle between the orientation vectors of two adjacent cells is greater than or equal to 0.97, the two cells are treated as in the same orientation and included in one crystallite, similar to the practice used in previous studies [53]. We also consider PBCs for the adjacent cells. In this way, we will be able to closely track the formation and growth of different crystallites during the cooling down process.

## 3. Results and Discussion

We first investigate the effect of different levels of nanoconfinement on the overall $X_{C}$ of the system. We then analyze the evolution of the crystallization process during cooling down by tracking different regions of the simulation box, which helps to better understand the differences in crystallization kinetics and when and how crystallite nucleation happens. Lastly, the effects of nanoconfinement under different cooling rates are also examined.

### 3.1. Effect of Nanoconfinement on $X_{C}$ and Crystallization Kinetics

**Figure 4:**
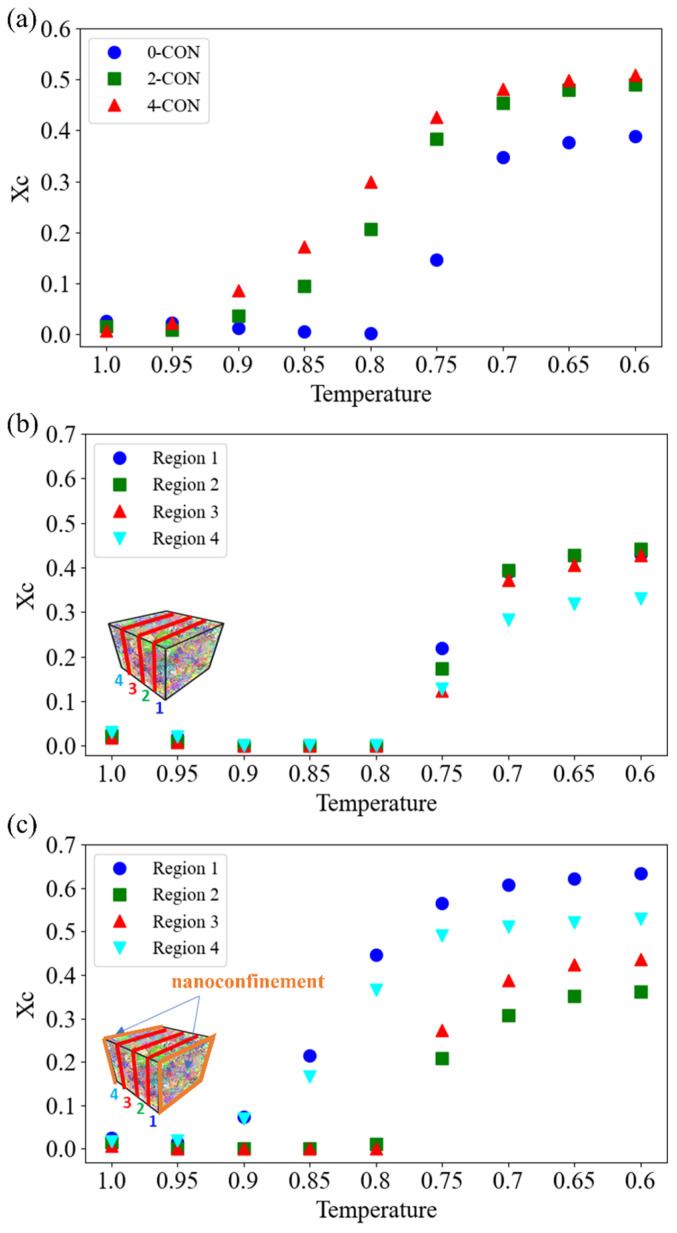
(**a**) Overall $X_{C}$ for the three levels of nanoconfinement considered herein. Region-dependent $X_{C}$ in the 0-CON (**b**) and 2-CON (**c**) cases.

Here, we first use the $p_{2}$ method to determine the $X_{C}$ of PVA under the three conditions shown in Figure 4a. We note that we use the decreasing temperature order in the *x*-axis to align with the cooling down process implemented in our simulations. As depicted in Figure 4a, the results show a notable augmentation in the final $X_{C}$ when the PVA is under nanoconfinement. Also, the 4-CON case shows an additional enhancement than the 2-CON case in the overall $X_{C}$ trend. Moreover, $X_{C}$ starts to rise significantly, beginning at T = 0.9 for the 4-CON case and T = 0.85 for the 2-CON case, much earlier than the bulk case for which $X_{C}$ starts to rise at T = 0.75. These onset temperatures typically indicate the beginning of crystallite formation or nucleation, which will be discussed further in Section 3.2. In terms of activation energy for crystallization, the rapid growth stage of $X_{C}$ tends to happen after the activation energies for both nucleation and growth have been achieved. Our results show that nanoconfinement facilitates the crystallization within PVA at an earlier stage of the cooling process. Despite the different crystallization onset temperatures, $X_{C}$ saturates at about T = 0.7 for all the cases.

To delve deeper, we divide up the cubic model into four even rectangular prism regions and compute $X_{C}$ of each region separately by comparing 0-CON (bulk, Figure 4b) and 2-CON (Figure 4c) cases. For the bulk PVA case in Figure 4b, all the four regions show increments of $X_{C}$ at the same temperature T = 0.75, consistent with the overall $X_{C}$ trend in Figure 4a. This indicates that the bulk PVA case exhibits homogenized crystallization kinetics overall. In contrast, for the 2-CON case depicted in Figure 4c, commencing at T = 0.9, the regions that are adjacent to the nanoconfinement show increments of $X_{C}$. But the regions that are further away from the nanoconfinement do not show crystallization at this temperature. This finding indicates that nanoconfinement can potentially decrease the activation energy for both nucleation and growth. Furthermore, the two middle regions show delayed initiation of crystallization until T = 0.8, similar to the bulk PVA case. Our results indicate possible heterogeneous crystallization kinetics induced by nanoconfinement, and we will look into this effect and the crystallite nucleation and growth processes in detail in the next section.

### 3.2. Effect of Nanoconfinement on Nucleation and Growth of Crystallites

**Figure 5:**
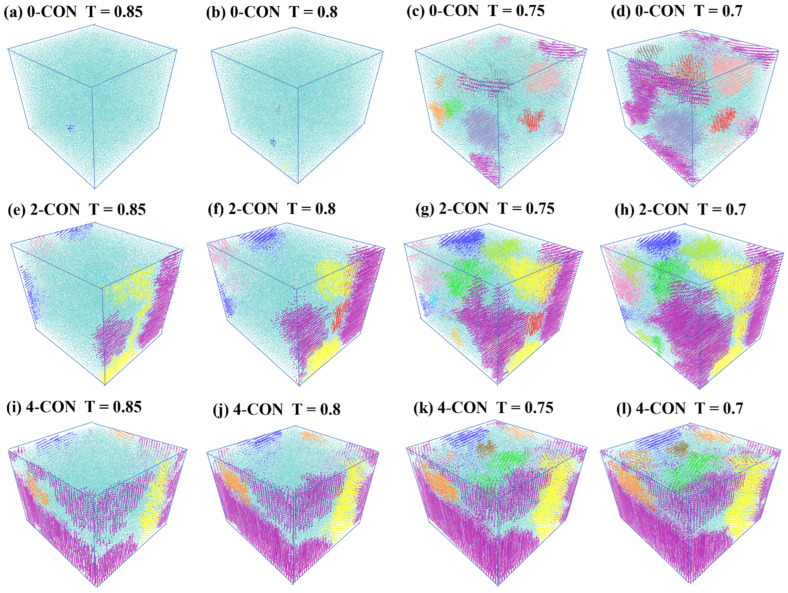
Visualization of the crystalline regions of PVA under different levels of nanoconfinement at various temperatures.

We label different crystallites identified with the nematic order method using different colored beads in Figure 5. Each color represents a distinct crystalline region. In this way, we can closely track the formation and growth of crystallites under different conditions. For the 0-CON case, no major crystallites are formed at temperatures higher than 0.8, whereas a major crystallite is defined as one consisting of at least 100 CG beads. At T = 0.75, several crystallites start to form randomly in the 0-CON model. From T = 0.75 to T = 0.7, we see the growth and coalescence of crystallites. For instance, the purple, green, and orange crystallites coalesce into one represented in purple from Figure 5c,d.

For nanoconfined cases, however, we observe that major crystallites already form at T = 0.85, indicating early nucleation and growth of crystallization. Also, crystallites in the 4-CON case are significantly larger than those in the 2-CON case. We also observe that crystallites initiate at the interfaces between PVA and the implicit walls that represent nanoparticle surfaces. As temperature decreases, we see significant growth of certain crystallites. These results further demonstrate that nanoconfinement can lead to reduction of activation energy of crystallization and thus result in early crystallization nucleation and growth.

**Figure 6:**
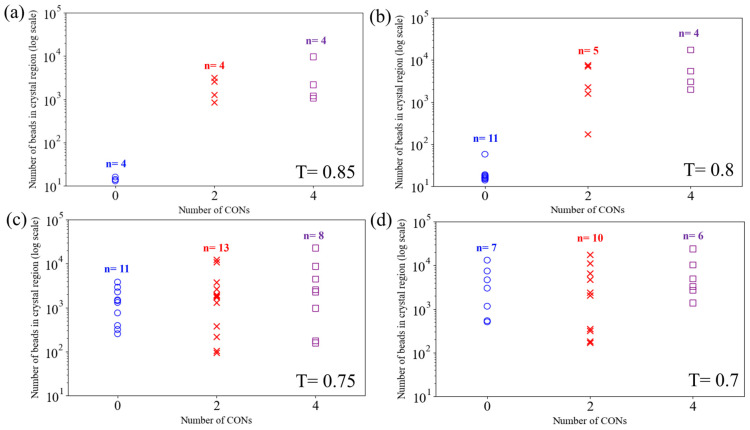
Comparison of numbers of the beads in each crystalline region under different levels of nanoconfinement at various temperatures T = 0.85 (**a**), T = 0.8 (**b**), T = 0.75 (**c**), and T = 0.7 (**d**).

To better understand the crystallite nucleation and coalesce processes, we track the number of CG beads in each identified crystallite in Figure 6.

In Figure 6, the blue circle points represent the counts of beads (i.e., the sizes) of each crystallite for the 0-CON case, while the red fork-shaped and purple square points depict the sizes of crystallites for 2-CON and 4-CON, respectively. We also mark the number of crystallites (n) for each case. In Figure 6a, at T = 0.85, the numbers of crystallites for the three cases are roughly the same, but the sizes of the crystallites differ significantly, with higher levels of nanoconfinement leading to much larger crystallites. Specifically, no major crystallites (count of beads smaller than 100) form in the 0-CON case. As shown in Figure 5e,i, major crystallites have already gone through nucleation and growth in 2-CON and 4-CON in regions next to interfaces, in contrast to the 0-CON case. At T = 0.8, there is mainly new crystallite formation or nucleation for the 0-CON case, as n shows a major increment, but crystallite growth is still dominant in 2-CON and 4-CON cases, as n stays roughly the same. From T = 0.8 to 0.75, the 0-CON case goes through the crystallite growth period, while, interestingly, 2-CON and 4-CON cases show new crystallite nucleation (increase in n). We believe the new crystallite nucleation happens in the internal regions away from the interfaces by referencing Figure 5. This observation indicates a second-stage crystallite nucleation process for the nanoconfined cases. This observation is attributed to the fact that the initial crystallization (nucleation and crystallite growth) process is predominantly influenced by the nanoconfinement, which occurs much earlier, and after most of the PVA chains in the vicinity of the interfaces have been crystallized, new nucleation sites start to form in regions further away from the interfaces. From T = 0.75 to T = 0.7, as the crystallization process comes to an end, crystallites begin coalescing in all three cases. We note that the 4-CON case shows the smallest number of crystalline regions, indicating that a higher level of nanoconfinement may further facilitate the merging of small crystalline regions into larger ones. The results shown in Figure 6 are in close accordance with the visualization of the crystalline regions shown in Figure 5.

Overall, our results show that the nanoconfinement introduced by the implicit rigid walls promotes early nucleation and growth of crystallite formation, as well as a heterogeneous nucleation process manifested by second-stage crystallite nucleation in regions further away from interfaces. Larger crystallite and higher crystallinity are generally observed with increasing levels of nanoconfinement. These results show agreement with a previous experimental study [68]. Nevertheless, we note that the effects of actual nanofillers on crystallization of semicrystalline polymers are more complex and can depend on multiple factors, including but not limited to nanofiller size and the interactions between nanofillers and polymers. We plan to look into these complex effects in our future work by modeling explicit nanofillers in a semicrystalline polymer matrix and studying their effects on the crystallization of semicrystalline polymers.

### 3.3. Effect of Nanoconfinement on Crystallization under Higher Cooling Rate

The crystallization behavior of PVA is greatly influenced by the cooling rate, as shown in a previous study [69]. Their experimental results showed that at a low cooling rate, PVA molecular chains have enough time to fold and align, thereby facilitating the generation of crystalline regions. We adjust the running steps accordingly to simulate the cooling process with different cooling rates. The corresponding running steps and cooling rate in our simulation are shown in Table 2. We note that, restrained by the limited computational results, the cooling rates adopted in the simulations are much higher than those experimental rates. Because of this, we do not observe much extended “lamellar” structure in semicrystalline PVA as observed in experiments. However, we believe the CG model can be leveraged to study the influence of cooling rate at a high cooling rate regime, and the Gibbs–Thomson-like relationships observed in the experiments can also be obtained using the present CG MD approach [50,56]. A recent study using the CG model showed that bulk polymers retain their amorphous configurations during cooling under very fast cooling rates [53].

**Figure 7:**
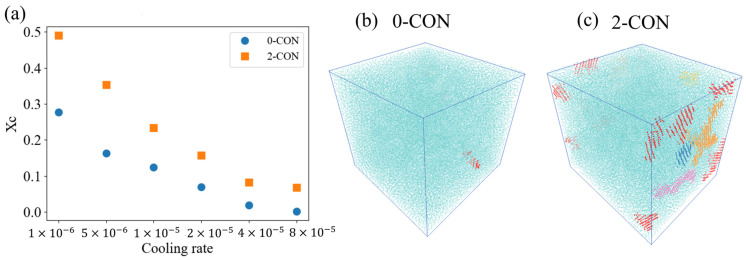
(**a**) $X_{C}$ vs. cooling rate for 0-CON and 2-CON cases. Crystallites formed under the highest cooling rate ($8\times 10^{-5}$) for 0-CON (**b**) and 2-CON (**c**) cases.

In this section, we aim to examine whether nanoconfinement still exhibits effects on polymer crystallization under such conditions. Under different cooling rates, $X_{C}$ of 0-CON and 2-CON cases at the temperature 0.6 are calculated and compared. The results are shown in Figure 7a, and the formed crystallites are also displayed in Figure 7b,c.

In Figure 7a, it is observed that the overall $X_{C}$ decreases with increasing cooling rate. In general, when the cooling rate increases, the crystallization process is farther away from thermodynamic equilibrium, and the response time for the polymer chains becomes too short to fold and eventually form crystallites [6,70]. While the tendencies under the two conditions are similar, the crystallinity of PVA under 2-CON is consistently larger than that of PVA under 0-CON at different cooling rates. Specifically, with the highest cooling rate of $8\times 10^{-5}$ in our simulation, the identified crystallites of PVA at T = 0.6 are displayed using colored beads in Figure 7b,c. In Figure 7b, only one small crystal region is observed in PVA as the temperature decreases to 0.6. However, in Figure 7c, under the same conditions, several crystallites are formed for the 2-CON. Moreover, majority of these crystallites nucleate at the interfaces, similar to these observed under lower cooling rates shown in Figure 5e. Therefore, we conclude that even under the very high cooling rate, nanoconfinement induced by interfaces still promotes the crystallization of PVA.

## 4. Conclusions

In this study, we have applied CG MD simulation to study the effect of nanoconfinement on the crystallization behaviors of PVA. We applied implicit walls that represent surfaces of nanoparticles or nanofillers to model PVA under different levels of nanoconfinement. By tracking region-dependent crystallinity and the specific crystallite nucleation and growth process with molecular scale to nanoscale details, we provide a fundamental delineation of the effect of nanoconfinement on the local crystallization behavior of the representative semicrystalline polymer. Specifically, overall $X_{C}$ and the average sizes of crystallites increase with the level nanoconfinement. The results of region-dependent $X_{C}$ and crystallization kinetics show that $X_{C}$ values in the regions closest to the interfaces are much higher than those in internal regions. Also, crystallites start to nucleate and grow in the regions next to the interfaces at higher temperature compared to those in the 0-CON case, which tend to nucleate randomly and grow within a small temperature range. Interestingly, our results further show that the nanoconfined cases also exhibit a heterogenous crystallization process manifested by a second-stage crystallite nucleation in regions further away from the interfaces. Finally, the effects of nanoconfinement on the crystallization process are compared for different cooling rates. Our finding shows that the overall $X_{C}$ decreases as the cooling rate increases. However, nanoconfinement shows a persistent enhancement effect on the overall $X_{C}$ and still promotes crystallite nucleation at interfaces with nanoparticles.

In the context of material design, our work can provide insights into the design of semicrystalline polymer-based nanocomposites. Particularly, the heterogeneous crystallization resulting from nanofiller-induced nanoconfinement manifested in this study can be leveraged to control the nano- to microscale structural evolution of semicrystalline polymer-based nanocomposites. In addition, our work serves as a guideline to better understand the structure–property relationship of PVA and other semicrystalline polymers. With a similar computational method, the crystallization process of polymer blends [71,72,73] and nanoparticle-reinforced polymer systems [74,75] could be further studied, providing fruitful topics for our future work.

## Figures and Tables

**Table 1:** The unit orientation vectors correspond to the 14 spherical neighbor domains.

Neighbor Domain Id	Unit Orientation Vector	Neighbor Domain Id	Unit Orientation Vector	Neighbor Domain Id	Unit Orientation Vector
1	(1,0,0)	6	(0,0,−1)	11	$\frac{1}{\sqrt{3}}(-1,-1,1)$
2	(−1,0,0)	7	$\frac{1}{\sqrt{3}}(1,1,1)$	12	$\frac{1}{\sqrt{3}}(-1,-1,-1)$
3	(0,1,0)	8	$\frac{1}{\sqrt{3}}(1,1,-1)$	13	$\frac{1}{\sqrt{3}}(1,-1,1)$
4	(0,−1,0)	9	$\frac{1}{\sqrt{3}}(-1,1,1)$	14	$\frac{1}{\sqrt{3}}(1,-1,-1)$
5	(0,0,1)	10	$\frac{1}{\sqrt{3}}(-1,1,-1)$		

**Table 2:** Different running steps and cooling rates selected here.

Time Step	Cooling Rate
$4\times 10^{7}$	$1\times 10^{-6}$
$8\times 10^{6}$	$5\times 10^{-6}$
$4\times 10^{6}$	$1\times 10^{-5}$
$2\times 10^{6}$	$2\times 10^{-5}$
$1\times 10^{6}$	$4\times 10^{-5}$
$0.5\times 10^{6}$	$8\times 10^{-5}$

## Data Availability

Data are contained within the article.
